# The Effects of Quiet Eye Training on Attention in Children with ADHD

**DOI:** 10.5114/jhk/168267

**Published:** 2023-07-06

**Authors:** Psotta Rudolf, Valtr Ludvík, Dostál Daniel

**Affiliations:** 1 Faculty of Physical Culture, Palacký University Olomouc, Olomouc, Czech Republic.; 2 College of Physical Culture and Sport PALESTRA, Prague, Czech Republic.; 3 Faculty of Arts, Palacky University Olomouc, Olomouc, Czech Republic.

**Keywords:** inattention, eye fixation, visuomotor control, intervention, childhood

## Abstract

Based on linkage between attention control, gaze and visuomotor control, previous studies suggested that quiet eye training (QET) could improve attention control. The aim of the study was to investigate the effects of QET-based visuomotor intervention on different aspects of attention in children with ADHD. Fifty seven children with ADHD aged 9–12 years, were tested on focused, tonic and phasic attention, as well as attention control during a throwing task analysed via eye tracking, in pre- and post-test sessions, six weeks apart. The QET group that underwent specific 5-week QET-based intervention significantly increased focused attention including its accuracy aspect. Reaction times (RTs) and intraindividual RT variability under non-alert and alert conditions as measures of tonic and phasic attention did not change over time in either group. In contrast with the control (CON) group, an extended total time of eye fixations during the pre-throw and throw phase was found in the QET group after the intervention. The results of this study suggest that focused attention in children with ADHD can be improved by a short-term QET-based visuomotor intervention. Future research needs to examine the effects of a longer QET-based intervention, or the intervention in combination with other cognitive training of attention.

## Introduction

Attention deficit/hyperactivity disorder (ADHD) is a neurodevelopmental disorder with a prevalence of approx. 5% among children (American Psychiatric Association, 2013). Based on data from 2015–2016, the prevalence of diagnosed ADHD in children aged 4 to 11 years was reported at 7.7% (Xu et al., 2018). Previous research has shown that inattention as a core feature of ADHD is associated with impairment in focused and sustained attention (Levy et al., 2018) and inhibition control (Crosbie et al., 2013). Attention control is closely related to gaze control and oculomotor behaviour (Vickers, 2009). Research has provided strong evidence that visual information which reaches the fovea via eye fixation is subsequently highly prioritised for further cortical processing (Raz and Buhle, 2006), and any shift in the gaze direction to a new location is always preceded by a shift in attention (Henderson, 2003; Mikicin et al., 2018). A strong association between eye movement and attention also ensues from the function of the oculomotor system in directing visual attention (Vickers, 2007). Since visual attention is a substantial mechanism for oculomotor behaviour (Henderson, 2003), eye movement events such as fixations, saccades and the recorded number of blinks are considered oculomotor markers of attention (Vickers, 2007).

Previous studies have brought evidence that children with ADHD display more atypical eye movements compared to typically developing children, highlighting their deficits in visual attention. ADHD children manifest difficulties in maintaining eye fixations and inhibition of automatic saccades when focusing on a target (Bucci et al., 2017), and impaired suppression of eye blinks (Fried et al., 2014).

Emerging from a bidirectional linkage between eye movements and attention during visually guided actions (Vickers, 2009), quiet eye (QE) training could potentially be useful for reducing attention deficits. QE training involves providing an individual with instructions and video-demonstrations of a highly skilled model of eye fixations during the execution of a targeting or interception task, with the aim of optimising his/her gaze control and in turn refining visuomotor performance (Vickers, 2007; Vine et al., 2014). QE training is based on the evidence that earlier and longer final eye fixation (called “quiet eye”) on a relevant cue immediately before the initiation of a movement, is indicative of superior visuomotor performance (Vickers, 2007; Vine et al., 2014). The explanation of the function of QE is that optical information received during this last fixation and processed into the central nervous system (CNS) is most critical for the motor programming of an action (Land, 2009; Vickers, 2007). Therefore, QE has been considered a measure of efficient visual attention control while performing a visually directed task (Vickers, 2007).

Short-term QE training used for throwing and catching in children with developmental coordination disorder (Miles et al., 2015a, 2015b; Wood et al., 2017) led to the prolongation of QE duration and earlier QE onset before the initiation of body movement. Those findings suggest that short-term QE training can lead to changes in gaze control with a close linkage to improved attention. However, there is no study on the effectiveness of QE training in attention control in children with ADHD. Therefore, the aim of the present study was to investigate the effects of QE training on different dimensions of attention in these children. Based on functional bidirectional linkage between gaze control, attention and visuomotor action (Vickers, 2009), we hypothesised that 5-week QE training focused on optimising eye fixations during performance of a visually guided motor task could be an effective method for ameliorating attention deficits in children with ADHD.

## Methods

### The Study Design and Participants

**Figure 1:**
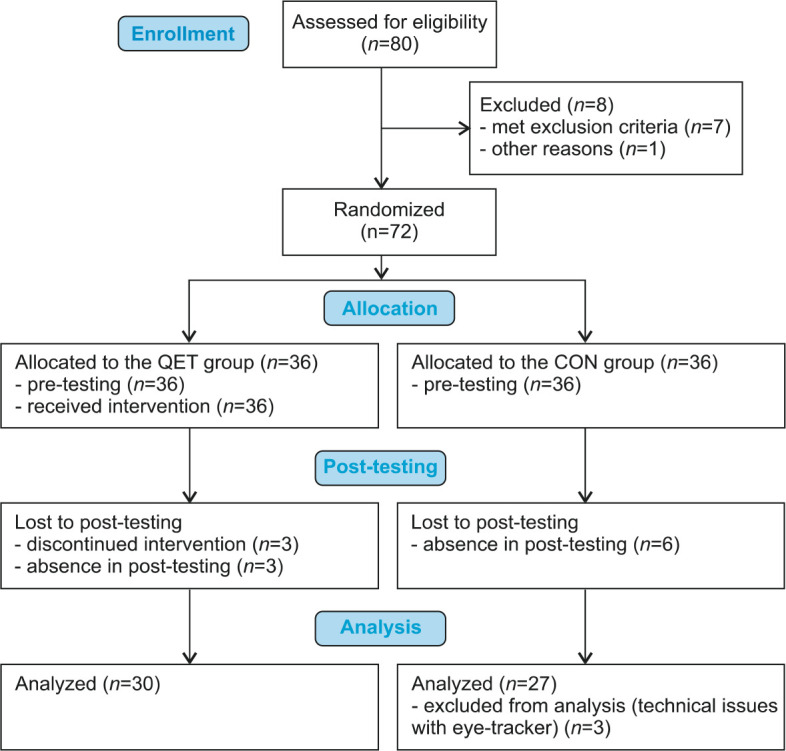
CONSORT 2010 flow diagram for study enrollment and participation.

The randomized double blind controlled trial included 72 children aged 9–12who met the diagnostic criteria for inattentive subtype (*n* = 66) and combined subtype of ADHD (*n* = 6) according to DSM-5 (American Psychiatric Association, 2013). Children with psychotic and conduct disorder, and physical, visual, hearing or neurological impairments were not recruited for the study. Participants were recruited from eleven public mainstream schools that provided education according to the national Framework Educational Program for Elementary Schools. Participants were assigned either to a QE training (QET) group or a control (CON) group by the technique of simple randomization with the use of computer-generated random numbers. The study was completed by 30 participants of the QET group (5 females), age 10.3 ± 1.1 years, two were left-handed, and 27 participants of the CON group (5 females), age 10.6 ± 1.0 years, three left-handed. Figure 1 presents the CONSORT flow diagram for study enrollment and participation. No significant differences were found between the groups in terms of age (*p* = 0.195), the percentage of males (*p* = 0.858), dominant hand (*p* = 0.562), ADHD subtype (*p* = 0.588) and medication (*p* = 0.473; 13 children medicated). Children and two instructors were not aware of the aim of the study. We obtained written informed consent from the legal guardians of all the participants. Research was performed in accordance with the Declaration of Helsinki of 1975. The study was approved by the ethics committee of the Faculty of Physical Culture, Palacký University Olomouc (approval code 312018; approval date: 20 February 2019).

### QE Visuomotor Training

The QET group underwent QE-based visuomotor training according to the protocol described by Miles et al. (2015b). Our training consisted in performance of targeting tasks (Table 1) accompanied with instructions and participant´s observation of a split-screen video with footage of the gaze and footage of body movement during a throwing action of a skilled model to emphasise the focusing of the participant´s gaze on a target. The split-screen video for each task (Table 1) was created from eye-tracker records including the location of a focal point within the visual field with a target, and from bodily movement records taken from the sagittal view of a very skilled 12-year-old individual during the performance of a targeting task. The gaze and motor videos were synchronised in the split-screen video and created at a playback speed of 100%, 50% and 25%.

**Table 1:** The tasks of quiet eye visuomotor training.

Training session	Major tasks
1^st^	Throwing the ball against a wall (2 m distance) and catching with two hands after bouncing
2^nd^	Overarm throwing a beanbag on one of three vertical openings of the construction (2 m distance)
3^rd^	Dart throwing from the distance of 2.37 m
4^th^	Underarm throwing a beanbag on one of three horizontal openings of the construction (2 m distance)
5^th^	Throwing a bolas tool to get caught on a vertical rung
	
	**Modifications of the tasks**
	Throwing from different distances and angles to a target
	The change of an alternative target (with exception of 1^st^ session)
	Using the non-preferred hand for throwing
	Different variability of changes for the modifications mentioned above

The QET group underwent five 35-min training sessions at the school of the given participant, one session a week (Table 1), all sessions were guided by a trained gradual instructor. Sessions were run according to the following protocol:

Step 1: Instruction and demonstration of the task by the instructor.

Step 2: Participant´s watching the split-screen video on a tablet with focusing of the gaze at the motor video at three playback speeds (approx. 90 s), then at the gaze video (approx. 90 s), accompanied with instructions to optimise the gaze before the initiation of throwing and during a throw.

Step 3: The participant summarised how to gaze in the pre-throw and the throw phases.

Step 4: The participant performed 30 practice trials of a task; after each 5 trials the instructor encouraged the participant to focus on a target while throwing.

Step 5: Participant´s watching the gaze video, and short instructions on the major points for optimising the gaze while throwing.

Step 6: The participant performed the final 20 practice trials of the task. After a 4-min rest interval, the participant performed various modifications of the task (Table 1) with the use of the 6-step training protocol presented above.

### Pre- and Post-Testing

The QET group underwent pre-tests one week before the 1^st^ training session and post-tests 7 days after the 5^th^ training session. The CON group performed post-tests 6 weeks after pre-tests. During the 6-week period between pre- and post-testing, children of the CON group participated in no special therapy or intervention that would be related to ADHD. Pre- and post-testing consisted of two neuropsychological tests and measurement of the eye movement pattern during a visuomotor targeting task as follows, always performed in counterbalanced order in each group.

### d2-R Test of Attention

The d2-R Test of Attention (Brickenkamp et al., 2014) is a standardized paper-and-pencil task that consists in searching of the target symbols (*d* with two dashes) mixed with distractors (*d* with one, three or four dashes and *p* with one to four dashes) that are located at 14 rows. The time for searching the target symbols is 20 s per row. Three variables were assessed: concentration performance (CP) as a measure of focused attention, the speed aspect of focused attention (PRZ), and the total percentage of commission and omission errors as a measure of the accuracy of focused attention (Err%). Construct and convergent validity as well as good to excellent reliability of the test were reported for children (Brickenkamp et al., 2014).

### Reaction Test of Alertness

To assess tonic and phasic attention, the standardized Reaction Test of Alertness (RTA) of the Vienna Test System (VTS) (Schuhfried, 2011) was used. This computer-based hand-eye simple reaction test consists of one set with 28 reactions to a stimulus (a yellow circle) (non-alert trials), and the second set of 28 reactions to a stimulus preceded by an acoustic warning signal (WS) lasting 500 ms, given 1000 ms before the appearance of the stimulus (alert trials). For the details see the literature by Schuhfried (2011) and Psotta et al. (2021). The following variables were assessed: mean RT in the non-alert trials and alert trials as a measure of tonic attention (RT-ton) and phasic attention (RT-pha), respectively, intraindividual coefficient of variation of RTs—CV RT-ton% and CV RT-pha%, respectively, as measures of the variability of arousal regulation, and the percentage of correct responses in all trials, CorrR-ton% and Corr-pha%, respectively. The average difference between RT-ton a RT-pha was used as a measure of alert effect (alerting score, AS). Incorrect responses were identified by VTS software. Excellent reliability, *r* = 0.965, for RT in the RTA was reported (Schuhfried, 2011).

### Measurement of the Eye Movement Pattern

To assess the effects of QE training on attention indicated by the participant’s eye fixation pattern, the throwing/catching task of the MABC-2 Test was performed (5 practice trials, 10 test trials) with the dominant hand by participants according to the MABC-2 Test manual (Henderson et al., 2007). For the test trials, the participant wore mobile SMI Eye Tracking Glasses 2w (ETG) (SMI, Teltow, Germany) to record eye movements (24 Hz) and the eye positions (60 Hz, gaze position accuracy of 0.5°). The ETG was connected to a mobile smart recorder (customised Samsung Galaxy S4 SmartPhone). Throwing arm movement was recorded by an external digital camera (50 Hz) placed at a distance of 3.5 m from the participant on the side of his/her throwing arm. To synchronize data on eye behaviour records and the arm movement record, the time point was optically marked by a flash of light concurrently recorded with the scene camera of the ETG and the external camera.

BeGaze 3.7 software (SMI, Teltow, Germany) was used to analyse the dataset of eye fixations, saccades and blinks prior to and during the throw phase of the task in each throwing trial.

**Figure 2:**
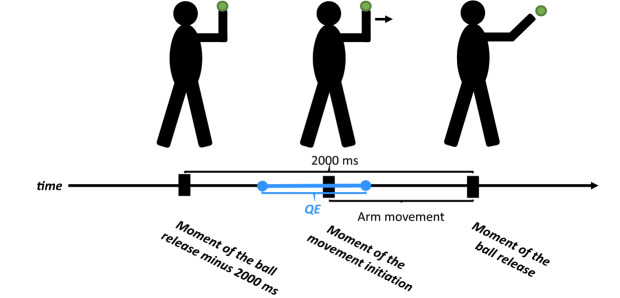
A schema of identification of the eye fixation variables related to three key action points.

Throwing arm movement was analysed using Dartfish 6.0 video analysis software (Dartfish, Fribourg, Switzerland). Three critical moments of arm movement were identified in the frame-by-frame manner procedure (Figure 2). Afterwards, based on the time synchronisation of the ETG data and the movement record, eye fixation data during separate throwing trials were extracted and the following variables were identified (Figure 2): (i) QE duration – defined as the last eye fixation within 1° of a “virtual” target on the wall before the initiation of movement (the onset of arm extension towards the target (Miles et al., 2015b). The starting position of the throwing arm was standardised to a 90° elbow angle; (ii) QE onset – the moment of movement initiation minus the onset of the QE fixation; (iii) QE offset – the difference between the offset of the QE fixation and the moment of movement initiation; (iv) the number of eye fixations within the interval of 2000 ms; (v) total time of eye fixations (TTEF) within the interval of 2000 ms. Arm movement time (MT_ARM_) was calculated as the time of ball release minus the time of the arm movement initiation (Figure 2).

### Data Analysis

Raw scores of the variables of the d2-R Test and the RTA (RT-ton, RT-pha and AS) were converted to age-based standard scores and T-scores, respectively. The results were analysed using a factorial 2 x 2 mixed-effects ANOVA with a fixed factor of Group (QET, CON) and Time (pre-test, post-test). The calculations were performed using the *lme4*, ver. 1.1-29 (Bates et al., 2015) and *emmeans*, ver. 1.7.3 (Lenth, 2022) libraries within the R environment. The effect size of interactions was quantified using ηp^2^ with the interpretation ηp^2^ = 0.01, 0.06, and 0.14 as small, medium, and large effect, respectively. The effects of time in the QET and CON groups were quantified with the effect size *d* calculated as the difference between the post- and pre-group mean divided by square root for a sum of residual variance of the model and variance of the proband random factor. Cohen’s *d* = 0.20, 0.50 and 0.80 was interpreted as low, medium, and large effects, respectively.

## Results

### Neuropsychological Tests

Table 2 presents descriptive statistics. According to the age-based norms of the d2-R Test of Attention (Brickenkamp et al., 2014), both groups achieved a very low or low baseline level in CP and Err% (Table 2). Both groups also showed a low level of both tonic and phasic alertness in the RTA when RT-ton and RT-pha corresponded to 14.5^th^ and 10.2^th^ percentile on average in the QET group, and 18.0^th^ and 14.3^th^ percentile on average in the CON group according to the age-based norms of the RTA (Schuhfried, 2011).

**Table 2:** Means and standard deviations in QET and CON groups.

		QET group		CON group	
Variable	Pre-test	Post-test	Pre-test	Post-test
*d2-R Test of Attention*				
CP (SS)	84.37 ± 15.43	95.67 ± 12.86	93.56 ± 8.87	98.33 ± 11.28
PRZ (SS)	97.00 ± 15.57	103.33 ± 13.87	98.59 ± 9.57	105.11 ± 9.61
Err% (SS)	86.53 ± 11.41	95.80 ± 14.18	93.74 ± 9.39	95.19 ± 12.61
*Reaction Test of Alertness (RTA)*				
RT-ton (T)	39.43 ± 6.80	37.27 ± 6.52	41.70 ± 8.06	39.52 ± 7.72
CV RT-ton(%)	15.57 ± 4.46	16.71 ± 4.85	15.54 ± 4.37	15.73 ± 4.20
CorrR-ton (n)	26.60 ± 1.22	26.07 ± 1.86	26.56 ± 1.89	26.52 ± 2.06
RT-pha (T)	37.20 ± 6.18	40.20 ± 9. 33	39.11 ± 8.26	42.59 ± 7.62
CV RT-pha(%)	18.88 ± 7.02	25.79 ± 13.87	19.94 ± 6.05	20.95 ± 9.10
CorrR-pha (n)	26.90 ± 1.32	26.27 ± 1.57	27.00 ± 1.98	26.67 ± 1.88
AS (T)	46.93 ± 10.73	53.73 ± 12.73	45.19 ± 12.39	54.78 ± 10.60
*Eye Fixation Pattern*				
Fixations (n)	3.74 ± 0.93	3.70 ± 0.94	4.18 ± 1.24	4.23 ± 1.06
TTEF (ms)	1428.13 ± 174.95	1539.57 ± 207.12	1526.70 ± 291.48	1484.63 ± 185.70
QE duration (ms)	856.87 ± 289.47	883.00 ± 262.06	640.74 ± 247.41	743.41 ± 303.51
QE onset (ms)	523.80 ± 235.94	596.53 ± 261.55	418.96 ± 206.46	468.52 ± 215.80
QE offset (ms)	439.20 ± 184.23	442.57 ± 153.38	287.96 ± 131.50	325.89 ± 174.45
*Catch Performance*				
Catch (n)	2.63 ± 2.62	4.07 ± 2.69	4.52 ± 2.90	4.96 ± 3.07
MT (ms)	614.03 ± 128.68	599.37 ± 140.97	576.63 ± 136.40	567.59 ± 134.61

Note: Err% – the number of errors; PRZ – the number of processed target characters; CP – concentration performance; AS – alert effect; RT-ton – mean reaction time in no-alert trials; CV RT-ton – coefficient of variation of RTs-ton; RT-pha – mean reaction time in alert trials; CV RT-pha – coefficient of variation of RTs-pha; CorrR-ton, CorrR-pha – a number of correct responses in no-alert and alert trials, respectively; TTEF – total time of eye fixations; QE – quiet eye; MT – arm movement time; SS – standard score; T – T-score

In the d2 Test of Attention, both groups achieved significantly higher post-values of CP than the pre-values, but with the high effect size (ES) in the QET group, in contrast to a moderate ES in the CON group (Table 3). The significant group x time interaction for CP confirmed significant improvement of CP in the QET group (Table 3). The QET group achieved a significantly lower Err% after QE training, in contrast to no significance in the CON group. Group x time interaction for Err% was significant (Table 3). The speed aspect of focused attention PRZ was improved across time in both groups, with no significant group x time interaction (Table 3).

**Table 3:** Comparison of effects in QET and CON groups.

Variable	Effect in QET group				Effect in CON group				Interaction Group × Time			
	*d*		*t*(57)	*p*	*d*		*t*(57)	*p*	*η* ^2^		*F*(1,57)	*p*
*d2-R Test of Attention*												
CP	0.92		5.37	< 0.001	0.39		2.16	0.035	0.07		4.56	0.037
PRZ	0.51		2.40	0.020	0.53		2.35	0.022	0.00		0.00	0.962
Err%	0.78		4.64	< 0.001	0.12		0.69	0.495	0.11		7.28	0.009
*Reaction Test of Alertness (RTA)*												
RT-ton (T)	−0.30		−1.53	0.131	−0.31		−1.47	0.148	0.00		0.00	0.993
CV RT-ton (%)	0.26		1.16	0.251	0.04		0.19	0.852	0.01		0.44	0.510
CorrR-ton (n)	−0.31		−1.26	0.213	−0.02		−0.08	0.934	0.01		0.65	0.423
RT-pha (T)	0.39		2.39	0.020	0.45		2.63	0.011	0.00		0.07	0.793
CV RT-pha (%)	0.73		2.94	0.005	0.11		0.41	0.684	0.05		2.98	0.090
CorrR-pha (n)	−0.38		−1.97	0.054	−0.20		−0.98	0.330	0.01		0.41	0.524
AS (T)	0.59		2.99	0.004	0.84		4.01	< 0.001	0.01		0.72	0.401
*Eye Fixation Pattern*												
Fixations (n)	−0.04		−0.23	0.822	0.05		0.33	0.746	0.00		0.15	0.696
TTEF (ms)	0.52		2.17	0.034	−0.20		−0.78	0.440	0.07		4.24	0.044
QE duration (ms)	0.10		0.47	0.639	0.38		1.76	0.084	0.02		0.91	0.345
QE onset (ms)	0.32		1.58	0.121	0.22		1.02	0.313	0.00		0.12	0.731
QE offset (ms)	0.02		0.09	0.928	0.24		0.97	0.338	0.01		0.41	0.525
*Catch Performance*												
Catch (n)		0.52	2.98	0.004		0.16	0.88	0.385		0.03	2.00	0.163
MT (ms)		−0.11	−0.69	0.493		−0.07	−0.40	0.688		0.00	0.03	0.856

Note: variables - see the note for Table 2; SS – standard score; T – T-score; d – effect size; t, F – test criteria; η^2^ – partial eta squared

The time factor (pre vs. post) was not significant for RT-ton in either group, similarly to group x time interaction for this variable. The RT-pha and AS variables significantly improved compared to the pre-values in both groups, with no significant group x time interaction (Table 3). No statistical significance was found for other variables of the RTA.

### Eye Fixation Pattern, Catch Performance and Arm Movement Time

TTEF was significantly prolonged from pre- to post-values in the QET group with moderate ES, while no significant change of TTEF was found in the CON group (Table 3). The effect of QE training on TTEF was indicated by the significant group x time interaction (Table 3).

No statistical significance was found for other variables of the eye movement pattern. Successful catches were increased across time in the QET group with a moderate ES, but not in the CON group (Table 3). No statistical significance was found for the movement time of the throwing arm.

## Discussion

The present study showed that QE training can lead to an enhancement in focused attention, indicated by positive changes in concentration performance (CP) and the number of errors Err% in the d2-R Test in the QET group. The major measure of focused attention CP is determined by both the accuracy (Err%) and speed (PRZ) of cognitive processing manifested during focused searching of target symbols (Brickenkamp et al., 2017). The variables Err% and PRZ represent unique cognitive qualities, in which very low correlations between them were confirmed (Brickenkamp et al., 2017). In our study, both groups achieved a higher speed of visual processing of symbols in the post-test, but only the QET group significantly increased the results in accuracy, i.e., reduced omission and commission errors. These findings suggest that the improved concentration performance in the QET group was mainly the result of the improved accuracy of focused cognitive activity, also including selective ignoring of distractors related to inhibitory control. It therefore appears that QE training emphasising visual attention on a target cue increased an individual’s ability to concentrate his/her attention on details in a visual field.

In contrast with focused attention, short-term QE training did not demonstrate any effectiveness for tonic attention as indicated by mean RT (RT-ton), intraindividual RT variability (CV RT-ton %) and the number of correct responses (CorrR-ton) under the non-alert condition of the RTA. Tonic attention is endogenously controlled alertness to response to external stimuli in the absence of an external cue in a top-down manner, and signifies the intensity dimension of attention (Keehn et al., 2013). One of the possible explanations for the lack of effect of QE training on tonic attention, besides the very short duration of training, could be the high genetic embedding of intrinsic attentional alertness (Finkel and Pedersen, 2014).

QE training did not influence phasic attention. Phasic attentional alertness is exogenously driven increased activation of the alerting network in a bottom-up manner when induced by a transient sensory input such as short sound, change in lighting, etc. (Keehn et al., 2013). As the alert effect is operationalised as the difference of RT under non-alert and alert conditions, the higher alert effect in the post-test observed in both groups was caused by shortened responses to the stimulus preceded by a WS, while RT under the non-alert condition did change. A previous study with typically developing children pointed out that during the middle period of childhood phasic attention is stabilised and alert effect is reduced (Psotta et al., 2021). The explanation could be that with developmental improvement in top-down controlled (tonic) alertness, children rely more on this attentional mode, and they can better exert cognitive control of attention (Lin et al., 1999). In contrast, younger children or children with impaired top-down control of attention could benefit from a WS in order to increase attentional alertness and attract their attention to a task more easily (Ishigami and Klein, 2015). We can therefore assume that the increased alert effect in our participants in the post-test was likely due to by their low level of top-down attention and in turn, the easier attracting of their attention by a WS when they became better accustomed to alert trials of the RTA in repeated testing.

Practising targeting tasks accompanied with instructions and video-demonstrations for the stimulation of visual attention on a relevant target led to the prolongation of TTEF during the pre-throw and throw phases. This prolongation of TTEF represents a longer time during which visual information entries the CNS and a reduction of total time of saccades and blinks that do not provide relevant optical input in the CNS (Vickers, 2007). As the number of eye fixations did not change across time, we can deduce that the average duration of one fixation was prolonged with QE training. The findings suggest that the QE training intervention led to better sustaining of eye fixations and less frequent distraction of attention while performing a visually directed motor task. The validity of the eye fixations during the throw action as a measure of visual attention control was supported by a similar strategy or technique of throws in the pre- and post-test, as indicated by non-significant changes in the arm movement time. The enhanced focused visual attention of the QET group, manifested during the targeting task, could correspond with the positive change in focused attention found in the d2-R Test.

Enhancement of catch performance in participants of the QET group after QE training (in contrast to the CON group) could be due to efficient neural programming of the throwing arm movement due to prolonged TTEF. It should be noted that in contrast with previous studies on QE training (Miles et al., 2015a, 2015b; Wood et al., 2017), we analysed eye behaviour during the pre-throw and throwing phases, not during the catch. We assumed that throwing the ball at the wall for catching on the rebound would be associated with a high demand to fix the eyes on a virtual location of a virtual target on the blank wall (Miles et al., 2015b). Thus, the level of top-down attention control can be better identified in comparison with a situation of easier evoking individual´s attention by moving the ball for catching. Previous studies have demonstrated that longer and more precise location of targeting QE fixation on a virtual target on a wall strongly predicted quick location and tracking the ball after it bounced (Wilson et al., 2013), earlier pre-catching QE (Wood et al., 2017), and catch performance (Miles et al., 2015a), explained by providing earlier information to prepare the interception attempt. Thus, the eye fixation pattern prior to and during a throw at a virtual target could be a more sensitive indicator of focused attention and its changes upon intervention.

A potential limitation of this study may be the inclusion of thirteen children receiving medication in order to alleviate cognitive or behavioural problems in connection with ADHD. According to Bucci et al. (2017), treatment with methylphenidate can reduce oculomotor abnormalities by means of adaptive strategies, which may incorporate the brain structure related to cognitive inhibition. As a result, in medicated children QE training may have not had such pronounced effects on attention, and performance in tests may have also been affected. Nonetheless, pre-tests showed a low to very low (abnormal) level of various dimensions of attention in the sample of children with ADHD. Furthermore, there was no significant difference between the number of children receiving medication in the QET and rol groups, and these children were a minority of the group in question (7 and 6 children, respectively). Despite the inclusion of the medicated children, the results of the study demonstrated a positive influence of the QE training intervention on concentration and visual focused attention. Incidentally, we were not authorised to adjust the children’s medication regimen.

Further, the results of the study relate to the immediate effects of visual motor training on attention in children with ADHD, and do not provide any evidence related to long-term changes of attention. Further research into non-pharmacological therapy of ADHD could be targeted in this direction.

## Conclusions

This is the first study to investigate the efficacy of short-term visuomotor intervention based on the paradigm of QE training in children with ADHD. The study demonstrated that practising visuomotor targeting tasks accompanied by providing participants with video-demonstrations of the gaze during targeting actions and instructions for optimising eye fixations on a target may be useful in order to stimulate focused attention in children with ADHD.
